# Oncology Update

**DOI:** 10.4103/0974-9233.65485

**Published:** 2010

**Authors:** Kaan Gündüz

**Affiliations:** Professor of Ophthalmology, Ankara University Faculty of Medicine, Ankara, Turkey

It gives me great pleasure to write this editorial for the special oncology issue of *MEAJO*. The topics selected for this issue represent developing diagnostic and therapeutic techniques in the field of ocular oncology. Some examples include fundus autofluorescence (FAF) and optical coherence tomography (OCT) imaging, exoresection and endoresection, photodynamic therapy, and intra-arterial chemotherapy that are increasingly being used in the field of ocular oncology. The purpose of this issue is to provide insight into these recent developments.

Turell and Singh undertook and in-depth review of the vascular tumors of the retina and choroid. Imaging features of these tumors including ultrasonography, angiography, and OCT are well illustrated in this article. Although vascular tumors of the retina and choroid constitute a histopathologically benign group of tumors, visual symptoms resulting from exudative retinal detachment remain a major source of visual disability. The current management of retinal and choroidal vascular tumors including photodynamic therapy, cryotherapy, and radiotherapy are comprehensively discussed by Turrell and Singh.

Materin and associates contributed an article on FAF and OCT findings in choroidal melanocytic lesions. They point out that overlying retinal pigment epithelial changes including drusen, lipofuscin, and fibrous metaplasia are better characterized by FAF, whereas OCT is more sensitive for the identification of subretinal and intraretinal fluid as well as atrophy, degeneration, and photoreceptor loss in the neurosensory retina. Since lipofuscin and subretinal fluid are among the risk factors predicting growth of choroidal nevus into a melanoma, their detection is of utmost importance. FAF and OCT are ancillary imaging methods that have helped to shape our treatment decisions. Furthermore, loss of photoreceptors on OCT may point out a more chronic, stable lesion, whereas an intact photoreceptor layer is indicative of a fast-growing possibly more aggressive-type of lesion.

Marr and colleagues were invited to write an article on the intra-arterial chemotherapy of retinoblastoma. They discuss skin changes following intra-arterial chemotherapy. A blanching erythematous and edematous patch was noted in 16% of the children who received intra-arterial chemotherapy. All patches of erythema spontaneously resolved within 3 months following completion of the intra-arterial chemotherapy. Intra-arterial chemotherapy of retinoblastoma remains an evolving treatment field. Hence as information and experience grows, our treatment paradigms will continue to evolve to incorporate potential benefits and address the complications and limitations of this procedure.

Gündüz and Bechrakis discussed the surgical resection of uveal melanomas. There are basically two surgical resection techniques: (1) Transscleral resection or “Exoresection” via a partial lamellar sclerouvectomy and “Endoresection” via a pars plana vitrectomy. While exoresection is more applicable to anteriorly located tumors with ciliary body and/or iris involvement, endoresection is more suitable for posteriorly located tumors without ciliary body involvement. In general, eyes containing posteriorly located choroidal melanoma with >7 mm thickness have a very dismal prognosis regarding long-term visual function, eye retention, and irradiation-induced side effects. By removing the tumor from the eye via endoresection, histopathologic and cytogenetic information of the tumor is available and significantly reduced incidence of ocular morbidity is encountered in the long term. However, both types of surgical resections are challenging procedures, bearing the risk of several complications. These are discussed in depth in this article.

Kandalam and associates reviewed the molecular pathology of retinoblastoma. Retinoblastoma develops from inactivation of both alleles of the *RB* 1 gene, a tumor suppressor gene. Other molecular defects involved in the RB tumor progression and mechanisms associated with inhibition of tumor cell apoptotic processes including p53, MDMX, and MDM2 genes are also discussed in this article. Retinoblastoma develops from heritable or sporadic mutations. Heritable mutations are present in all the retinal and body cells whereas sporadic mutations occur in only the retinal cells. Patients with heritable mutations not only require more frequent examinations under anesthesia, but also are faced with the risk of developing different cancers. Secondary nonocular cancers associated with external beam radiotherapy and various cancers in the context of RB1 cancer predisposition syndrome occur preferentially in heritable retinoblastoma patients. Therefore, molecular pathology for retinoblastoma is an extremely important issue.

Finally, I thank the Editor-in-Chief of *MEAJO* and the editorial team of *MEAJO* for their collaboration, help, and understanding in the preparation of this issue. I also extend my sincere thanks to all our contributing authors from various countries for their efforts and time in the preparation of the articles in this issue. As the *MEAJO* family, we will continue to improve our journal to the best of our capabilities to make it a consistently referenced source in PubMed.

